# The genome sequence of the angle shades moth, *Phlogophora meticulosa *(Linnaeus, 1758)

**DOI:** 10.12688/wellcomeopenres.17757.1

**Published:** 2022-03-14

**Authors:** Douglas Boyes, Peter W.H. Holland, University of Oxford and Wytham Woods Genome Acquisition Lab, Darwin Tree of Life Barcoding collective, Wellcome Sanger Institute Tree of Life programme, Wellcome Sanger Institute Scientific Operations: DNA Pipelines collective, Tree of Life Core Informatics collective, Darwin Tree of Life Consortium

**Affiliations:** 1 UK Centre for Ecology and Hydrology, Wallingford, Oxfordshire, UK; 2 Department of Zoology, University of Oxford, Oxford, UK

**Keywords:** Phlogophora meticulosa, angle shades, genome sequence, chromosomal, Lepidoptera

## Abstract

We present a genome assembly from an individual female *Phlogophora meticulosa *(the angle shades; Arthropoda; Insecta; Lepidoptera; Noctuidae). The genome sequence is 539 megabases in span. The majority of the assembly, 95.17%, is scaffolded into 31 chromosomal pseudomolecules, with the Z sex chromosome assembled. Some unassigned scaffolds are identified as belonging to the W chromosome based on half-depth coverage and comparison to other Noctuidae W chromosomes. The mitochondrial genome was also assembled and is 15.4 kilobases in length.

## Species taxonomy

Eukaryota; Metazoa; Ecdysozoa; Arthropoda; Hexapoda; Insecta; Pterygota; Neoptera; Endopterygota; Lepidoptera; Glossata; Ditrysia; Noctuoidea; Noctuidae; Hadeninae; Phlogophora; *Phlogophora meticulosa* (Linnaeus, 1758) (NCBI:txid875884).

## Background

*Phlogophora meticulosa* (angle shades) is a delicately-patterned moth with characteristically 'wrinkled' wings that do not lie in a flat plane; the unusual wing form contributes to camouflage amongst dead leaves and may also influence flight through permitting deformation ( Wootton, 2020). The species can be found across most of Europe, and throughout the UK and Ireland where it is more frequent in the south. The species is migratory and occurs commonly in parks, gardens, woodland edges and coastal areas. In the UK, adults are generally observed from March to November in two flight periods, with the peak of each flight period having shifted earlier by about 3 weeks over the past 30 years ( Randle *et al.*, 2019). The larvae of *P. meticulosa* are polyphagous and have variable colouration ranging from bright green to dark brown, with colour being controlled by food plant in early larval instars and by multiple, as yet unidentified, genetic loci at later larval instars ( Majerus, 1978). The genome of *P. meticulosa* was sequenced as part of the Darwin Tree of Life Project, a collaborative effort to sequence all of the named eukaryotic species in the Atlantic Archipelago of Britain and Ireland. Here we present a chromosomally complete genome sequence for *P. meticulosa*, based on one female specimen from Wytham Woods, Oxfordshire, UK.

## Genome sequence report

**Figure 1.**
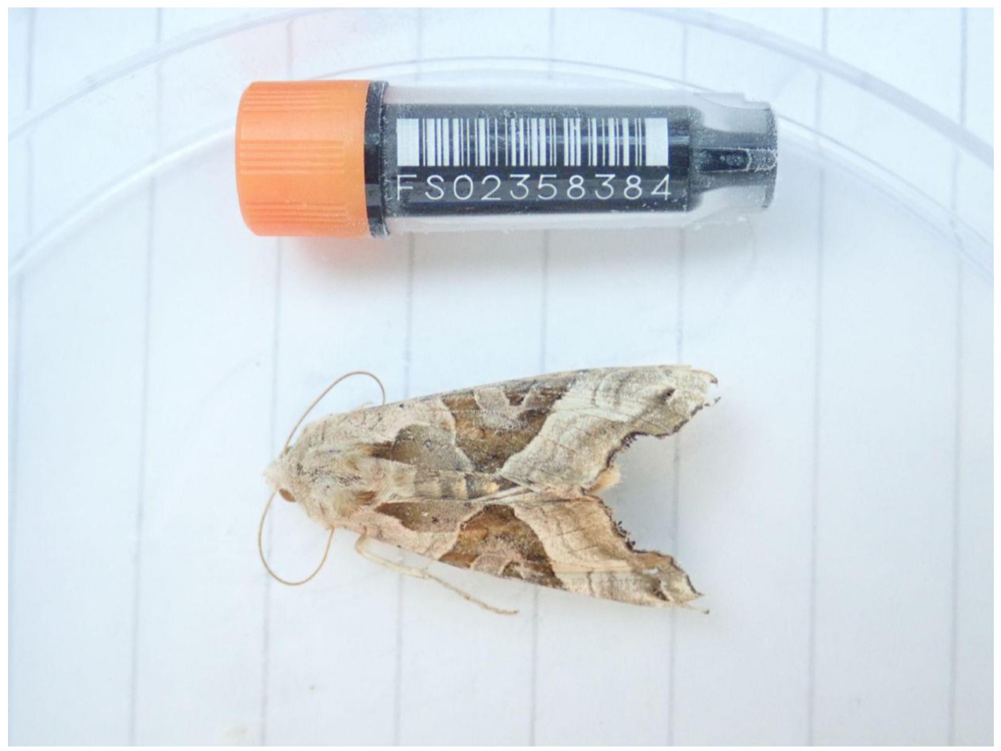
Image of the ilPhlMeti2 specimen taken prior to preservation and processing. Specimen shown next to FluidX storage tube, 43.9 mm in length.

The genome was sequenced from a single female *P. meticulosa* (ilPhlMeti2; Figure 1) collected from Wytham Woods, Oxfordshire, UK (latitude 51.772, longitude -1.338). A total of 45-fold coverage in Pacific Biosciences single-molecule long reads (N50 12 kb) and 77-fold coverage in 10X Genomics read clouds were generated. Primary assembly contigs were scaffolded with chromosome conformation Hi-C data. Manual assembly curation corrected 10 missing/misjoins, reducing the scaffold number by 14.81%, and increasing the scaffold N50 by 2.73%. 

**Figure 2.**
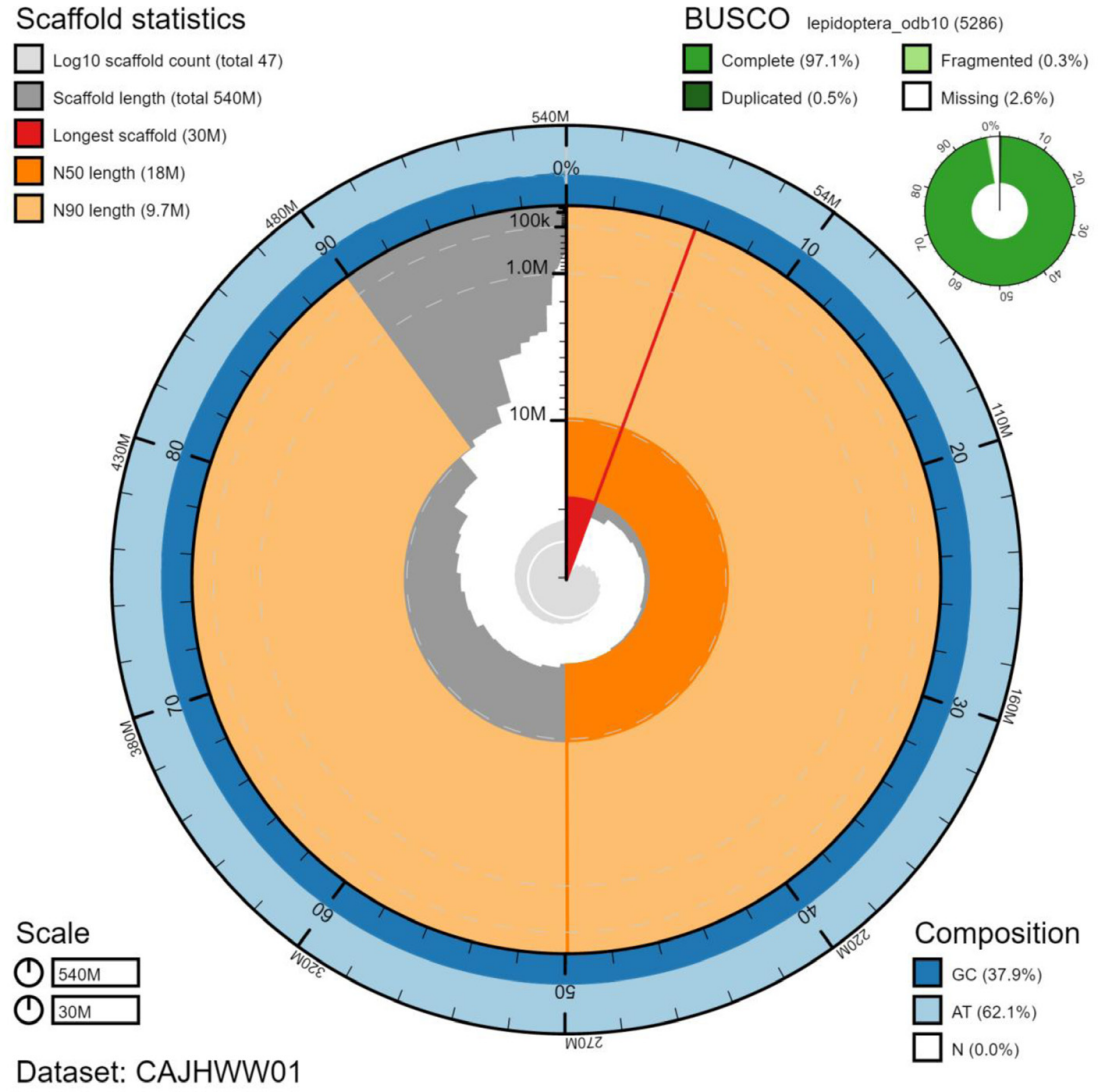
Genome assembly of *Phlogophora meticulosa*, ilPhlMeti2.1: metrics. The BlobToolKit Snailplot shows N50 metrics and BUSCO gene completeness. The main plot is divided into 1,000 size-ordered bins around the circumference with each bin representing 0.1% of the 538,659,867 bp assembly. The distribution of chromosome lengths is shown in dark grey with the plot radius scaled to the longest chromosome present in the assembly (30,333,309 bp, shown in red). Orange and pale-orange arcs show the N50 and N90 chromosome lengths (18,098,236 and 9,704,819 bp), respectively. The pale grey spiral shows the cumulative chromosome count on a log scale with white scale lines showing successive orders of magnitude. The blue and pale-blue area around the outside of the plot shows the distribution of GC, AT and N percentages in the same bins as the inner plot. A summary of complete, fragmented, duplicated and missing BUSCO genes in the lepidoptera_odb10 set is shown in the top right. An interactive version of this figure is available at https://blobtoolkit.genomehubs.org/view/ilPhlMeti2.1/dataset/CAJHWW01/snail.

The final assembly has a total length of 539 Mb in 47 sequence scaffolds with a scaffold N50 of 18 Mb ( Table 1). Of the assembly sequence, 95.2% was assigned to 31 chromosomal-level scaffolds, representing 30 autosomes (numbered by sequence length), and the Z sex chromosome ( Figure 2– Figure 5; Table 2). Some unassigned scaffolds are compatible with belonging to the W chromosome based on half-depth read coverage, high repeat content and comparison with other Noctuidae W chromosomes. The assembly has a BUSCO ( Manni *et al.*, 2021) completeness of 97.1% (single 96.7%, duplicated 0.5%) using the lepidoptera_odb10 reference set (n=5286). While not fully phased, the assembly deposited is of one haplotype. Contigs corresponding to the second haplotype have also been deposited.

**Table 1.** Genome data for *Phlogophora meticulosa*, ilPhlMeti2.1.

*Project accession data*	
Assembly identifier	ilPhlMeti2
Species	*Phlogophora meticulosa*
Specimen	ilPhlMeti2
NCBI taxonomy ID	NCBI:txid875884
BioProject	PRJEB41949
BioSample ID	SAMEA7520192
Isolate information	Female, head/abdomen/thorax
*Raw data accessions*	
PacificBiosciences SEQUEL II	ERR6594496
10X Genomics Illumina	ERR6002732-ERR6002735
Hi-C Illumina	ERR6002736
PolyA RNA-Seq Illumina	ERR6002737, ERR6787418
*Genome assembly*	
Assembly accession	GCA_905147745.1
*Accession of alternate haplotype*	GCA_905147725.1
Span (Mb)	539
Number of contigs	61
Contig N50 length (Mb)	17
Number of scaffolds	47
Scaffold N50 length (Mb)	18
Longest scaffold (Mb)	20
BUSCO* genome score	C:97.5%[S:96.5%,D:1.0%],F:0.4%,M:2.1%,n:1658

**Figure 3.**
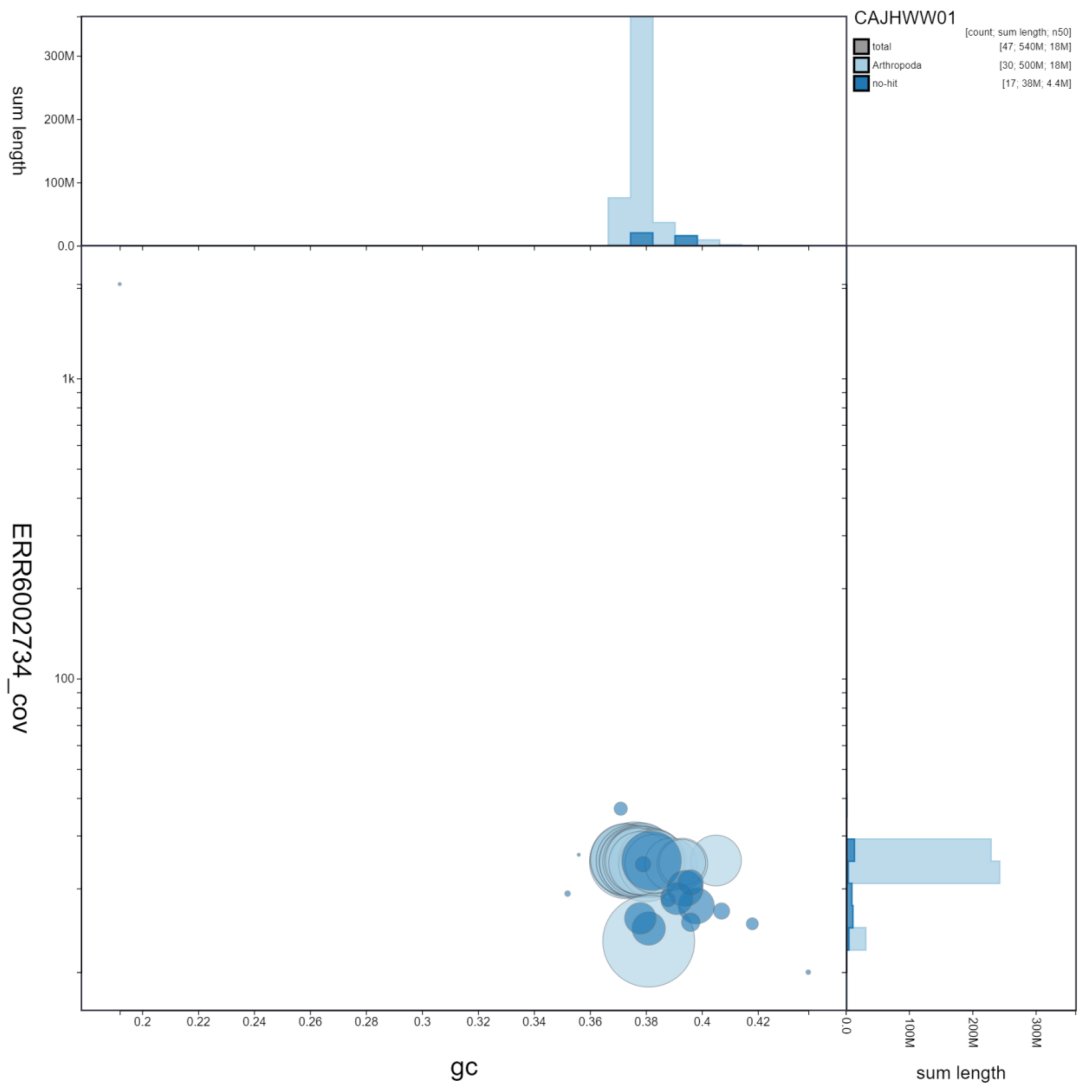
Genome assembly of *Phlogophora meticulosa*, ilPhlMeti2.1: GC coverage. BlobToolKit GC-coverage plot. Scaffolds are coloured by phylum. Circles are sized in proportion to scaffold length. Histograms show the distribution of scaffold length sum along each axis. An interactive version of this figure is available at https://blobtoolkit.genomehubs.org/view/ilPhlMeti2.1/dataset/CAJHWW01/blob.

**Figure 4.**
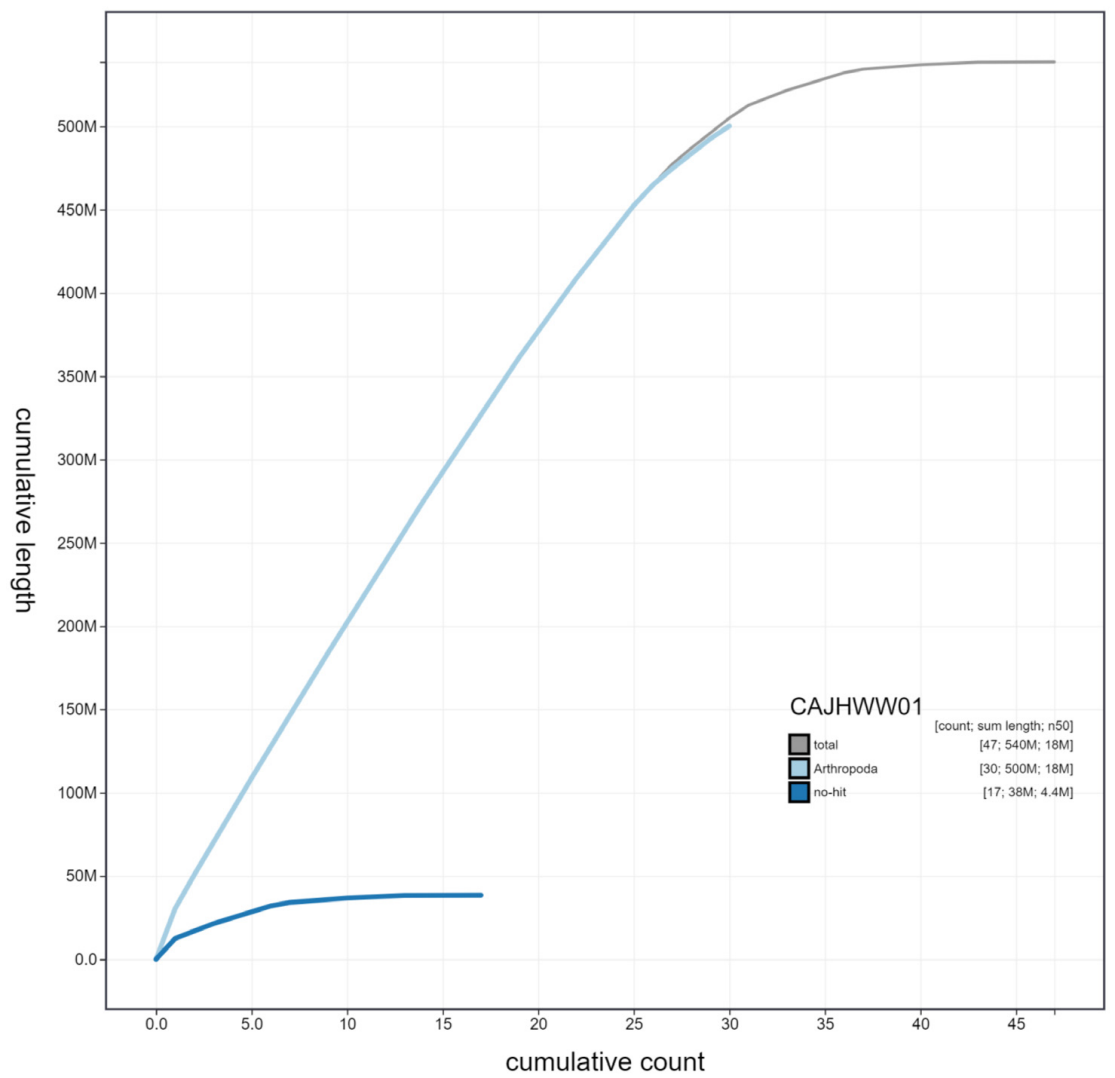
Genome assembly of *Phlogophora meticulosa*, ilPhlMeti2.1: cumulative sequence. BlobToolKit cumulative sequence plot. The grey line shows cumulative length for all scaffolds. Coloured lines show cumulative lengths of scaffolds assigned to each phylum using the buscogenes taxrule. An interactive version of this figure is available at https://blobtoolkit.genomehubs.org/view/ilPhlMeti2.1/dataset/CAJHWW01/cumulative.

**Figure 5.**
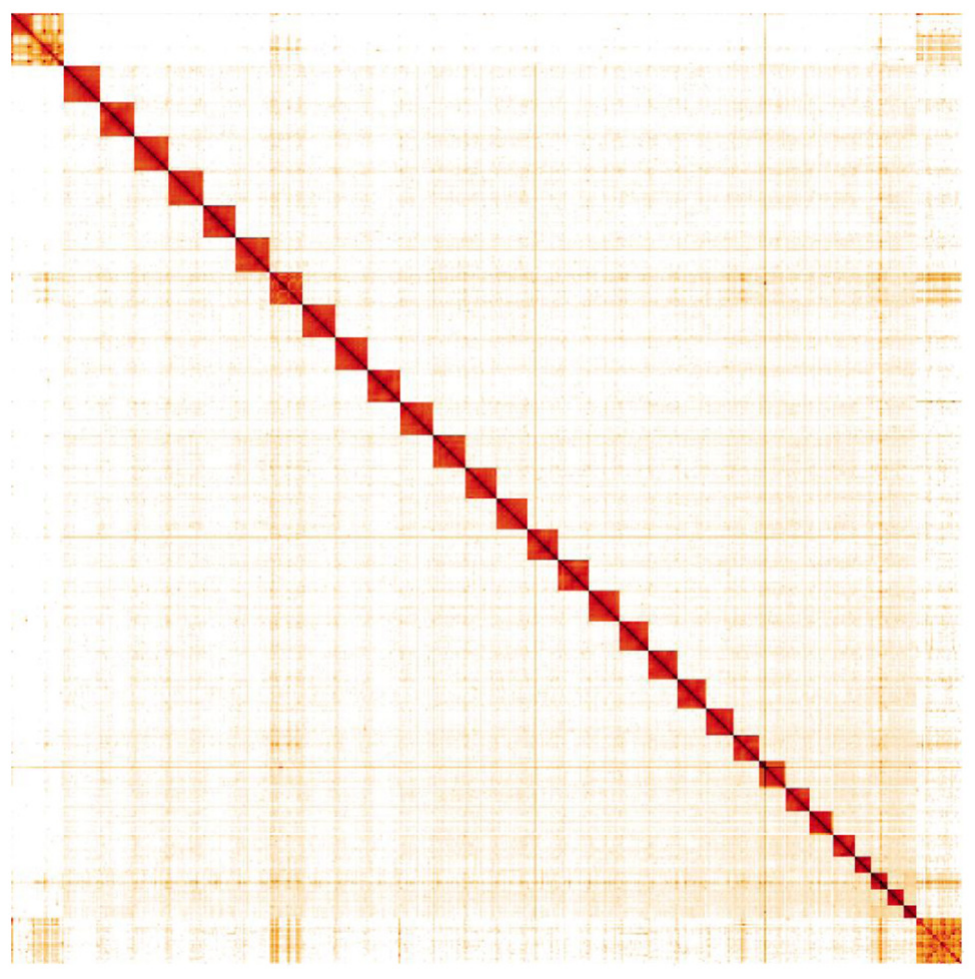
Genome assembly of *Phlogophora meticulosa*, ilPhlMeti2.1: Hi-C contact map Hi-C contact map of the ilPhlMeti2.1 assembly, visualised in HiGlass.

**Table 2.** Chromosomal pseudomolecules in the genome assembly of *Phlogophora meticulosa*, ilPhlMeti2.1.

INSDC accession	Chromosome	Size (Mb)	GC%
LR990518.1	1	20.34	37.6
LR990519.1	2	19.51	37.8
LR990520.1	3	19.41	37.3
LR990521.1	4	19.14	37.4
LR990522.1	5	18.92	37.8
LR990523.1	6	18.86	37.3
LR990524.1	7	18.80	38
LR990525.1	8	18.58	37.5
LR990526.1	9	18.45	37.7
LR990527.1	10	18.40	37.8
LR990528.1	11	18.17	37.3
LR990529.1	12	18.16	37.5
LR990530.1	13	18.10	37.6
LR990531.1	14	17.62	37.6
LR990532.1	15	17.29	37.7
LR990533.1	16	17.26	37.8
LR990534.1	17	17.07	38
LR990535.1	18	16.88	37.8
LR990536.1	19	16.09	38.2
LR990537.1	20	15.90	38.2
LR990538.1	21	15.50	37.7
LR990539.1	22	15.02	38.2
LR990540.1	23	14.52	38.3
LR990541.1	24	14.19	37.8
LR990542.1	25	12.45	38.2
LR990543.1	26	12.28	38.3
LR990544.1	27	9.70	38.9
LR990545.1	28	9.12	40.5
LR990546.1	29	8.99	39.3
LR990547.1	30	7.61	39.3
LR990517.1	Z	30.33	38.1
LR990548.1	MT	0.02	19.5
-	Unplaced	25.97	39

## Methods

### Sample acquisition and nucleic acid extraction

One female *P. meticulosa* (ilPhlMeti2) and one *P. meticulosa* of unknown sex (ilPhlMeti1) were collected from Wytham Woods, Oxfordshire, UK (latitude 51.772, longitude -1.338) by Douglas Boyes, UKCEH, using a light trap. The specimen was identified by the same individual and preserved on dry ice.

DNA was extracted from head/thorax tissue at the Wellcome Sanger Institute (WSI) Scientific Operations core from the whole organism using the Qiagen MagAttract HMW DNA kit, according to the manufacturer’s instructions. RNA was extracted from head/thorax and abdomen tissue of ilPhlMeti1 in the Tree of Life Laboratory at the WSI using TRIzol (Invitrogen), according to the manufacturer’s instructions. RNA was then eluted in 50 μl RNAse-free water and its concentration RNA assessed using a Nanodrop spectrophotometer and Qubit Fluorometer using the Qubit RNA Broad-Range (BR) Assay kit. Analysis of the integrity of the RNA was done using Agilent RNA 6000 Pico Kit and Eukaryotic Total RNA assay.

### Sequencing

Pacific Biosciences HiFi circular consensus and 10X Genomics Chromium read cloud sequencing libraries were constructed according to the manufacturers’ instructions. Poly(A) RNA-Seq libraries were constructed using the NEB Ultra II RNA Library Prep kit. Sequencing was performed by the Scientific Operations core at the Wellcome Sanger Institute on Pacific Biosciences SEQUEL II (HiFi), Illumina HiSeq X (10X) and Illumina HiSeq 4000 (RNA-Seq) instruments. Hi-C data were generated from abdomen tissue of ilPhlMeti2 using the Arima v1 Hi-C kit and sequenced on HiSeq X.

### Genome assembly

Assembly was carried out with Hifiasm ( Cheng *et al.*, 2021); haplotypic duplication was identified and removed with purge_dups ( Guan *et al.*, 2020). One round of polishing was performed by aligning 10X Genomics read data to the assembly with longranger align, calling variants with freebayes ( Garrison & Marth, 2012). The assembly was then scaffolded with Hi-C data ( Rao *et al.*, 2014) using SALSA2 ( Ghurye *et al.*, 2019). The assembly was checked for contamination and corrected using the gEVAL system ( Chow *et al.*, 2016) as described previously ( Howe *et al.*, 2021). Manual curation ( Howe *et al.*, 2021) was performed using gEVAL, HiGlass ( Kerpedjiev *et al.*, 2018) and Pretext. The mitochondrial genome was assembled using MitoHiFi ( Uliano-Silva *et al.*, 2021), which performed annotation using MitoFinder ( Allio *et al.*, 2020). The genome was analysed and BUSCO scores generated within the BlobToolKit environment ( Challis *et al.*, 2020). Table 3 contains a list of all software tool versions used, where appropriate.

**Table 3.** Software tools used.

Software tool	Version	Source
Hifiasm	0.12	Cheng *et al.*, 2021
purge_dups	1.2.3	Guan *et al*. 2020
SALSA2	2.2	Ghurye *et al*. 2019
longranger align	2.2.2	https://support.10xgenomics.com/genome-exome/software/pipelines/latest/advanced/other-pipelines
freebayes	1.3.1-17-gaa2ace8	Garrison and Marth, 2012
MitoHiFi	1.0	https://github.com/marcelauliano/MitoHiFi
gEVAL	0.1.x	Chow *et al*. 2016
HiGlass	1.11.6	Kerpedjiev *et al*. 2018
BlobToolKit	2.6.2	Challis *et al*. 2020

### Ethics/compliance issues

The materials that have contributed to this genome note have been supplied by a Darwin Tree of Life Partner. The submission of materials by a Darwin Tree of Life Partner is subject to the Darwin Tree of Life Project Sampling Code of Practice. By agreeing with and signing up to the Sampling Code of Practice, the Darwin Tree of Life Partner agrees they will meet the legal and ethical requirements and standards set out within this document in respect of all samples acquired for, and supplied to, the Darwin Tree of Life Project. Each transfer of samples is further undertaken according to a Research Collaboration Agreement or Material Transfer Agreement entered into by the Darwin Tree of Life Partner, Genome Research Limited (operating as the Wellcome Sanger Institute), and in some circumstances other Darwin Tree of Life collaborators.

## Data availability

European Nucleotide Archive: Phlogophora meticulosa (angle shades). Accession number PRJEB42141; https://identifiers.org/ena.embl/PRJEB42141.

The genome sequence is released openly for reuse. The *P. meticulosa* genome sequencing initiative is part of the Darwin Tree of Life (DToL) project. All raw sequence data and the assembly have been deposited in INSDC databases. The genome will be annotated using the RNA-Seq data and presented through the Ensembl pipeline at the European Bioinformatics Institute. Raw data and assembly accession identifiers are reported in Table 1.

## Author information

Members of the University of Oxford and Wytham Woods Genome Acquisition Lab are listed here: https://doi.org/10.5281/zenodo.5746938.

Members of the Darwin Tree of Life Barcoding collective are listed here: https://doi.org/10.5281/zenodo.5744972.

Members of the Wellcome Sanger Institute Tree of Life programme are listed here: https://doi.org/10.5281/zenodo.6125027.

Members of Wellcome Sanger Institute Scientific Operations: DNA Pipelines collective are listed here: https://doi.org/10.5281/zenodo.5746904.

Members of the Tree of Life Core Informatics collective are listed here: https://doi.org/10.5281/zenodo.6125046.

Members of the Darwin Tree of Life Consortium are listed here: https://doi.org/10.5281/zenodo.5638618.
