# Supplementary material for: The genome sequence of the angle shades moth, Phlogophora meticulosa (Linnaeus, 1758)
Source: Wellcome Open Res. 2022 Mar 14;7:89. [Version 1] doi: 10.12688/wellcomeopenres.17757.1 (PMC9608255; doi:10.12688/wellcomeopenres.17757.1)
Supplement: Peer review history

# Peer review: Reviewer response for version 1

**DOI:** 10.21956/wellcomeopenres.19653.r50941

**Published:** 2022-06-22

**Authors:** Jing Zhang

**Affiliations:** 1 Eugene McDermott Center for Human Growth and Development, University of Texas Southwestern Medical Center, Dallas, TX, USA

The authors assembled a high-quality chromosome-level genome of angle shades moth with various sequencing techniques, including PacBio, 10X Genomics and Hi-C. The manuscript is well written. The only question I have is if authors also used 10X Genomics read data for scaffolding or generating contigs. From the manuscript, I noticed that 10X Genomics read data is only used for polishing the assembly and correcting small polymorphisms. But the advantage of 10X Genomics read data over common Illumina pair-end short-read data is to provide long-range genomic information and to help scaffold contigs. Conventional Illumina short read sequencing data is enough for fixing base errors (SNV/Indel)

Are sufficient details of methods and materials provided to allow replication by others?

Yes

Is the rationale for creating the dataset(s) clearly described?

Yes

Are the datasets clearly presented in a useable and accessible format?

Yes

Are the protocols appropriate and is the work technically sound?

Yes

Reviewer Expertise:

Genomics, Computational biology

I confirm that I have read this submission and believe that I have an appropriate level of expertise to confirm that it is of an acceptable scientific standard.

# Peer review: Reviewer response for version 1

**DOI:** 10.21956/wellcomeopenres.19653.r49197

**Published:** 2022-04-01

**Authors:** Sara J. Oppenheim

**Affiliations:** 1 Sackler Institute for Comparative Genomics,, American Museum of Natural History, New York, NY, USA

This is a very nicely done sequencing project and the assembly stats are excellent. The MS will be improved by adding a more detailed justification of why the sequencing was carried out and what sort of interesting biological phenomena might be explored. Such information would enhance the intro and allow for some discussion/conclusions.

 The figures are largely useless and all of them are hard to read because the legends are super tiny.

 Some further details are needed regarding the treatment of samples and which samples were used for which approach.

 These issues are detailed in the annotated version of the MS that I have provided here.

Are sufficient details of methods and materials provided to allow replication by others?

Partly

Is the rationale for creating the dataset(s) clearly described?

Partly

Are the datasets clearly presented in a useable and accessible format?

Yes

Are the protocols appropriate and is the work technically sound?

Yes

Reviewer Expertise:

Evolutionary biology; quantitative genetics; genome sequencing, assembly, and annotation; ecological adaptation in the Lepidoptera; bioinformatics.

I confirm that I have read this submission and believe that I have an appropriate level of expertise to confirm that it is of an acceptable scientific standard.
